# Complete genome sequence of Deltapapillomavirus 4 (bovine papillomavirus 2) from a bovine papillomavirus lesion in Amazon Region, Brazil

**DOI:** 10.1590/0074-02760160047

**Published:** 2016-04

**Authors:** Cíntia Daudt, Flavio RC da Silva, Samuel P Cibulski, Matheus N Weber, Fabiana Q Mayer, Ana Paula M Varela, Paulo M Roehe, Cláudio W Canal

**Affiliations:** 1 Universidade Federal do Rio Grande do Sul, Faculdade de Veterinária, Laboratório de Virologia, Porto Alegre, RS, Brasil; 2 Universidade Federal do Acre, Centro de Ciências Biológicas e da Natureza, Rio Branco, AC, Brasil; 3 Fundação Estadual de Pesquisa Agropecuária, Instituto de Pesquisas Veterinárias Desidério Finamor, Laboratório de Biologia Molecular, Eldorado do Sul, RS, Brasil; 4 Universidade Federal do Rio Grande do Sul, Departamento de Microbiologia, Imunologia e Parasitologia, Laboratório de Virologia, Porto Alegre, RS, Brasil

**Keywords:** papillomavirus, bovine, BPV2, complete genome

## Abstract

The complete genome sequence of bovine papillomavirus 2 (BPV2) from Brazilian Amazon Region was determined using multiple-primed rolling circle amplification followed by Illumina sequencing. The genome is 7,947 bp long, with 45.9% GC content. It encodes seven early (*E1*, *E2*,*E4*, *E5*, *E6*,*E7*, and *E8*) and two late (*L1* and *L2*) genes. The complete genome of a BPV2 can help in future studies since this BPV type is highly reported worldwide although the lack of complete genome sequences available.

Papillomaviruses (PVs) are small, oncogenic, highly epitheliotropic viruses with marked tropism for squamous epithelia (Bravo & Felez-Sanchez 2015). The genome of PVs is a circular molecule of double stranded DNA of about 8 kb, which bears one of the slowest evolutionary rates among viruses (Rector et al. 2007).

Fifteen bovine papillomavirus (BPV) types have been recognised to date (BPV1-BPV15) and are classified into four genera and five species. BPV infections have been reported worldwide; among these, BPV2 has been reported as one of the most prevalent types (Hatama et al. 2011, Roperto et al. 2013, Araldi et al. 2014). The BPV2 is assigned to the Deltapapillomavirus genus species 4. Apart from causing infections in the original host (cattle), this virus type has been recovered from lesions in other species, such as the equines and in buffaloes (Corteggio et al. 2013, Kumar et al. 2015).

There are few studies on the genetic diversity and distribution of BPV in Brazil. Despite this paucity of data, it is known that the BPV2 is the most detected virus in Brazilian cattle (Batista et al. 2013, Araldi et al. 2014, da Silva et al. 2015). In order to expand the knowledge on the genetic diversity of the BPV2, the complete genome sequencing of an autochthonous BPV2 from the Brazilian Amazon Region is described.

A rolling circle amplification (RCA) was applied to 100 ng of total DNA isolated from a papilloma lesion as previously described (Dezen et al. 2010, Rijsewijk et al. 2011). Neoplastic tissue was comprised by exophytic papillomatous, epithelium proliferation, and well-differentiated cells, marked acanthosis, koilocytes, increased amounts of granules in the granular layer, and keratohyalin granules. Libraries were prepared with Nextera DNA sample preparation kit (Illumina) using the RCA products and sequenced in an Illumina MiSeq System with MiSeq reagent kit v2 300 cycle. Reads were assembled into contigs using SPAdes 3.6 and compared to sequences in the GenBank nucleotide and protein databases using BLASTn/BLASTx. The Geneious software was used for open reading frame (ORF) predictions and genome annotations.

A total of 27,764 reads were produced, of which 8,116 were related to BPV2 (average reads length 111 nt). One full-length circular contig related to BPV2 was identified and annotated (mean coverage 92). The circular genome was named BPV2 BRA/09RO12. It spans 7,947 bp, with a 45.9% GC content (Figure). The genome potentially encodes seven early (*E1*, *E2*,*E4*, *E5*, *E6*, *E7*, and *E8*) and two late ORFs (*L1* and*L2*). A 934 bp noncoding region (NCR) is located between the*L1* and *E6* ORFs (Figure).

**Figure:**
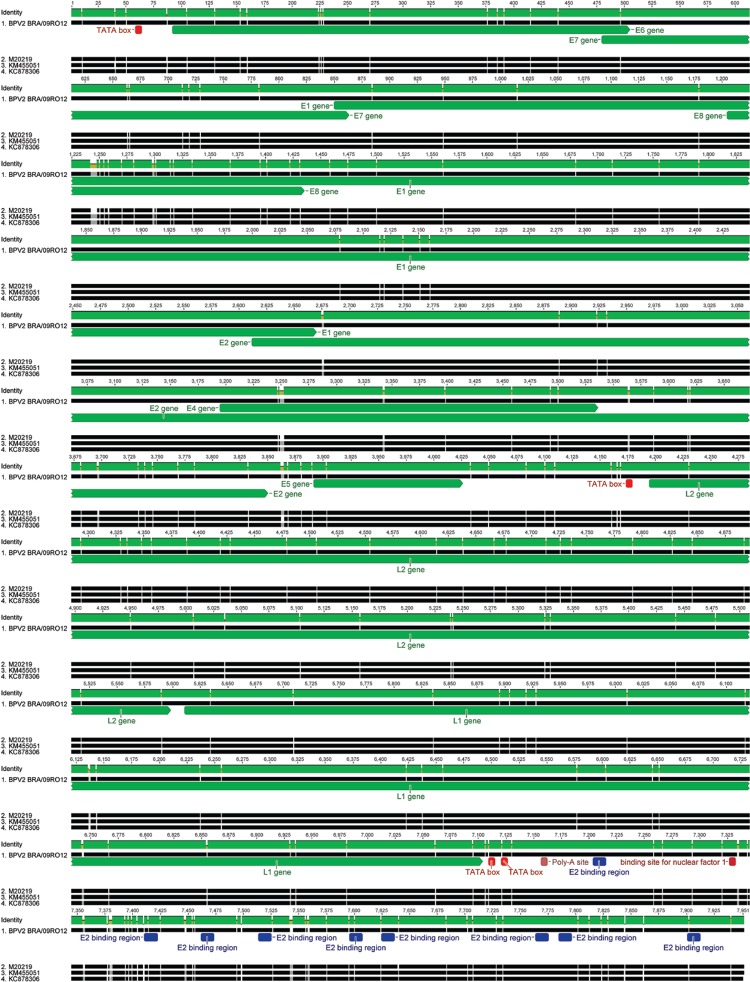
Nucleotide alignment of bovine papillomavirus 2 (BPV2) BRA/09RO12 with the complete genomes of BPV2 available in GenBank. Putative coding regions of BPV2 BRA/09RO12 for early (*E1*, *E2*,*E4*, *E5*, *E6*,*E7*, and E8) and late proteins (L1 and L2) are marked by arrows.

The gene *E1* encodes the largest viral protein (with helicase function), which contains 606 amino acids; the adenosine 5’-triphosphate (ATP)-binding site (GPPNTGKS) of the ATP-dependent helicase is present in the carboxy-terminal part of*E1* (Titolo et al. 1999). The putative E6 protein exhibits two conserved zinc-binding domains of CX_2_CX_29_CX_2_C (Lehoux et al. 2009). The E5 protein shows a leucine-rich profile, while E7 exhibits a proline-rich profile. The NCR contains nine consensus palindromic E2-binding sites (ACCN_6_GGT), three putative TATA boxes (TATAAA) of E6 promoter, and the polyadenylation site (AATAAA) for *L1* and *L2*transcripts (Zheng & Baker 2006, de Villiers & Gunst 2009).

The sequence reported here (BRA/09RO12) shares a high degree of nucleotide identity among BPV2 genomes available at GenBank (97.7% with a North American BPV2 reference genome M20219 and ~98.5% with recently sequenced Chinese strains KC878306 and KM455051) (Figure). As expected, most differences in the nucleotide sequences were concentrated in the NCR and in the *E8* gene (Garcia-Vallve et al. 2006). Double stranded viruses show the slowest evolutionary rates among viruses (Sanjuan et al. 2010). As example, two BPV1 sequences reported in Sweden and in United States of America more than 30 years apart displayed 99.89% nucleotide identity, not different from the standing genetic variation of this virus (Ahola et al. 1983).

The complete genome of BPV2 BRA/09RO12 is the first complete BPV2 recovered from Brazilian cattle reared in the Amazon Region. It reveals a high degree of identity (> 97%) with previously published BPV2 reported elsewhere, thus confirming the worldwide prevalence of such virus type. This sequence is expected to assist future studies on genetic comparisons and characterisation of PV genomes.

*Nucleotide sequence accession* - The complete genome sequence of BPV2 strain BRA/09RO12 is available in GenBank under the accession KU674833.
